# A Unified Framework for Inattention Estimation From Resting State Phase Synchrony Using Machine Learning

**DOI:** 10.3389/fgene.2021.728913

**Published:** 2021-09-23

**Authors:** Xun-Heng Wang, Lihua Li

**Affiliations:** Institute of Biomedical Engineering and Instrumentation, Hangzhou Dianzi University, Hangzhou, China

**Keywords:** predictive models, inattention, feature selection, regression algorithms, phase synchrony

## Abstract

Inattention is one of the most significant clinical symptoms for evaluating attention deficit hyperactivity disorder (ADHD). Previous inattention estimations were performed using clinical scales. Recently, predictive models for inattention have been established for brain-behavior estimation using neuroimaging features. However, the performance of inattention estimation could be improved for conventional brain-behavior models with additional feature selection, machine learning algorithms, and validation procedures. This paper aimed to propose a unified framework for inattention estimation from resting state fMRI to improve the classical brain-behavior models. Phase synchrony was derived as raw features, which were selected with minimum-redundancy maximum-relevancy (mRMR) method. Six machine learning algorithms were applied as regression methods. 100 runs of 10-fold cross-validations were performed on the ADHD-200 datasets. The relevance vector machines (RVMs) based on the mRMR features for the brain-behavior models significantly improve the performance of inattention estimation. The mRMR-RVM models could achieve a total accuracy of 0.53. Furthermore, predictive patterns for inattention were discovered by the mRMR technique. We found that the bilateral subcortical-cerebellum networks exhibited the most predictive phase synchrony patterns for inattention. Together, an optimized strategy named mRMR-RVM for brain-behavior models was found for inattention estimation. The predictive patterns might help better understand the phase synchrony mechanisms for inattention.

## Introduction

Estimating personalized cognitive or behavioral scores from neuroimaging is an interesting yet challenging topic nowadays (Rosenberg et al., 2016; Shen et al., 2017; Yoo et al., 2017; Rosenberg et al., 2018; Sui et al., 2020). The individual brain-age, Intelligence Quotient (IQ), attention, as well as personality can be estimated either from structural or functional MRI using machine learning (Zhao et al., 2019; Cai et al., 2020; Lin et al., 2020; Munsell et al., 2020; Niu et al., 2020). Among those brain-behavior models, predicting individual attention from neuroimaging has drawn a significant amount of research interests (Rosenberg et al., 2016, 2018; Yoo et al., 2017). Attention is a key function in psychology. Attention is also a significant feature for diagnosis of ADHD (Xiao et al., 2016; Zhao et al., 2018; Wang et al., 2018a,b). Inattention can lead to dysfunction of memory, learning, and other important cognitive tasks (Brown et al., 2009; Fassbender et al., 2011; Vaidya et al., 2020). Before the present time, the inattention scores were always estimated using clinical scales, which were subjective measures reported by participants (Zhang et al., 2005). Furthermore, the neural mechanisms of inattention are still unclear to date. Therefore, it is of great interest to build predictive models for inattention using resting state fMRI.

The predictive models for inattention estimations contain three parts. One important component of a predictive model is the input features. Currently, most of the raw features for inattention estimations were based on linear functional connectivity (Rosenberg et al., 2016; Yoo et al., 2017). The nonlinear complexity (i.e., phase synchrony) remained unknown (Wang et al., 2017). Another important component is the regression algorithms. The well-established connectome-based predictive modeling (CPM) for inattention estimation was based on multi-linear regression (Shen et al., 2017). The comparisons of performance of different regression algorithms remain largely unexplored (Yoo et al., 2017; Sui et al., 2020). The third component is the model validation procedure. So far, most of the predictive models were evaluated using leave-one-out cross validation. Although several studies validated their models using two independent datasets, the N-fold cross validations might also be beneficial for inattention estimation (Scheinost et al., 2019).

In addition, different preprocessing steps (i.e., global signal regression (GSR), data scrubbing) might have impacts on the brain connectivity (Li et al., 2019a). Although the benefits of GSR for resting fMRI are still under debate, previous studies found that GSR might enhance the brain-behavior relationships (Murphy et al., 2009; Wong et al., 2012; Li et al., 2019a). The data scrubbing or volume censoring methods also have impacts on functional connectivity features (Yan et al., 2013; Parkes et al., 2018; Li et al., 2019b; Lindquist et al., 2019). Therefore, different preprocessing steps should be considered in the brain-behavior regression tasks. So far, the effects of different preprocessing procedures on estimation of inattention using phase synchrony remain unclear.

In this paper, we aimed to apply a unified framework to estimate the personalized inattention from resting state phase synchrony. First, a cohort of participants with both inattention scores and resting state fMRI datasets were obtained from the ADHD-200 database. Then, the resting state fMRI datasets were preprocessed using different strategies that were with or without GSR or scrubbing. Third, the regional signals were obtained from the normalized images. Fourth, phase synchrony was derived as input for the regression tasks. Fifth, the inattention scores were estimated using different regression algorithms. Finally, the regression models were analyzed using 100 runs of 10-fold cross validations. The impacts of different preprocessing strategies on the regression tasks are compared in the results section. The predictive patterns are discussed in the discussion section.

## Materials and Methods

### Participants and MRI Protocols

Participants in this study were obtained from the ADHD-200 database. To be consistent with previous studies, the samples from the Peking University were selected as subjects. There were 95 ADHD and 126 healthy controls. Each participant signed the consent form that was approved by the ethics committee of Peking University. The inattention scores were measured using the ADHD rating scales. For each participant, a high-resolution T-1 weighted anatomical MRI and a sequence of resting state fMRI datasets (TR=2s, 235 volumes) were acquired using a Siemens 3T MRI scanner. The detailed information of MRI parameters could be found at the website of ADHD-200.^1^

### Data Preprocessing

The anatomical MRI were skull-stripped, segmented, and nonlinearly deformed to standard space. The resting state fMRI was normalized using the following procedures: dropped the first five volumes, slice-timing, motion correction, skull-stripped, nuisance signal regression, temporal filtering (0.01–0.1Hz), scrubbing, spatial normalization. Specially, an artifactual volume was marked with frame-wise displacement >0.5mm or DVARS value =1. The forward volume and backward volume were also marked as artifactual scan points. The detailed information of data preprocessing could be found in previous works (Wang et al., 2017, 2018b). After preprocessing, the regional time-courses were extracted using a previously well-established brain atlas that consisted of 268 functional nodes (Shen et al., 2013).

### Phase Synchrony

The phase synchrony is a bivariate complexity measure with nonlinear properties. The phase synchrony has been widely applied in neuroscience as an alternative feature for conventional functional connectivity. One advantage of phase synchrony was the nonnegative property. Another advantage was the nonlinear property. The phase synchrony could be obtained using the following steps: (1) get the instantaneous phases of each time-signal using Hilbert transform; (2) unwarp the instantaneous phases; (3) get the instantaneous phase differences between each pair of time-signals; (4) discard the artifactual instantaneous phase differences if scrubbing was applied on preprocessing steps; and (5) compute the mean phase coherence as phase synchrony index (Sun and Small, 2009; Sun et al., 2012).

### Regression Models

The minimum-redundancy maximum-relevancy (mRMR) features (Ding and Peng, 2005) were selected using the praznik package.^2^ A number of features were detected based on significant correlations with inattention (*p*<0.05). First, the number of significant inattention-correlated features (*p*<0.05) was obtained in each cross-validation. Second, the numbers of features were obtained after 100 runs of 10-fold cross-validations. Finally, the mean value of numbers of features was calculated for the mRMR procedure. In addition, the classical correlation coefficients method was also applied to select features (*p*<0.05). The predictive power of inattention-correlated features with *p*<0.05 and r>0 was analyzed additionally. The features selected by the covariance between inattention and phase synchrony were analyzed with the number of features the same as that of the mRMR. The regression models were solved using six algorithms: the support vector regression (SVR), the partial least squares (PLS), the relevance vector machine (RVM), the ridge regression (RR), the elastic net (ENET), and the least absolute shrinkage and selection operator (LASSO). In this study, the SVR algorithm was carried out using the svm() function in e1071 package.^3^ The PLS algorithm was carried out using the pls () function in the texir package.^4^ The RVM algorithm was carried out using the rvm() function in kernlab package,^5^ which automatically solved the sigma parameter. The RR, ENET and LASSO algorithms were carried out using the glmnet() function in the glmnet package^6^ with alpha=0, 0.5, and 1, respectively. The six algorithms used their default parameters in the R packages for comparisons of cross-validations. The CPM algorithm was carried out additionally using the MATLAB toolbox.^7^ Furthermore, the parameters were fine-tuned for the regression algorithms using the caret package.^8^ The RR, lasso, and ENET were analyzed using the glmnet model, which fine-tuned the alpha and lambda parameters. The PLS algorithm was analyzed using the pls model, which fine-tuned the number of component parameter. The support vector machine algorithm was analyzed using the svmLinear model, which fine-tuned the cost parameter.

### Evaluations

**Figure 1:**
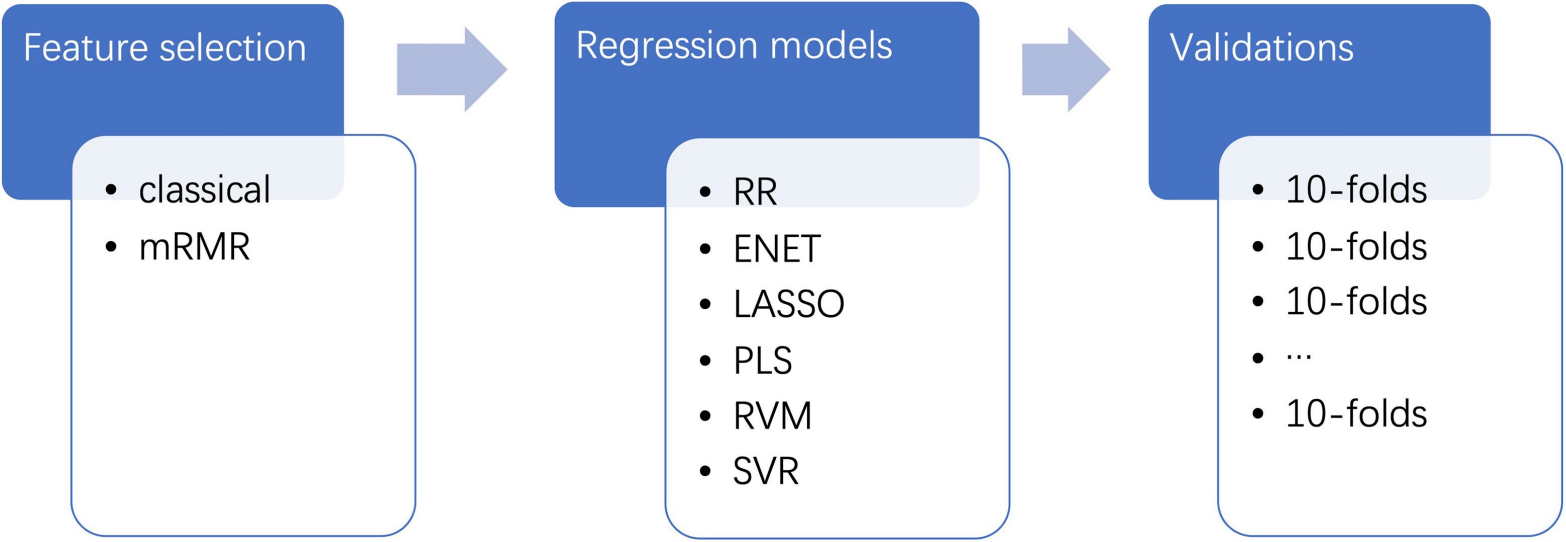
Pipelines for the predictive models. The raw features of phase synchrony are firstly selected by two feature selection methods. Then, the selected features are trained and tested using several regression algorithms. Finally, the predictive models are validated using 100 runs of 10-fold cross-validations.

In this paper, 100 runs of 10-fold cross-validations were applied on the regression tasks. For each run, the original samples were divided into 10 folds. For each fold, nine folds of training samples and a fold of testing samples were applied to build predictive models. The outputs of 10 folds were joined together to match with the original inattention scores. The performance of the regression models was evaluated by correlation coefficients, which were computed using the 1,000 times of permutations test. The values of *p* were analyzed using the RVAideMemoire package.^9^ The pipeline for the feature selection, regression, and validation procedures could be found in Figure 1.

## Results

### Performance of Predictive Models

**Figure 2:**
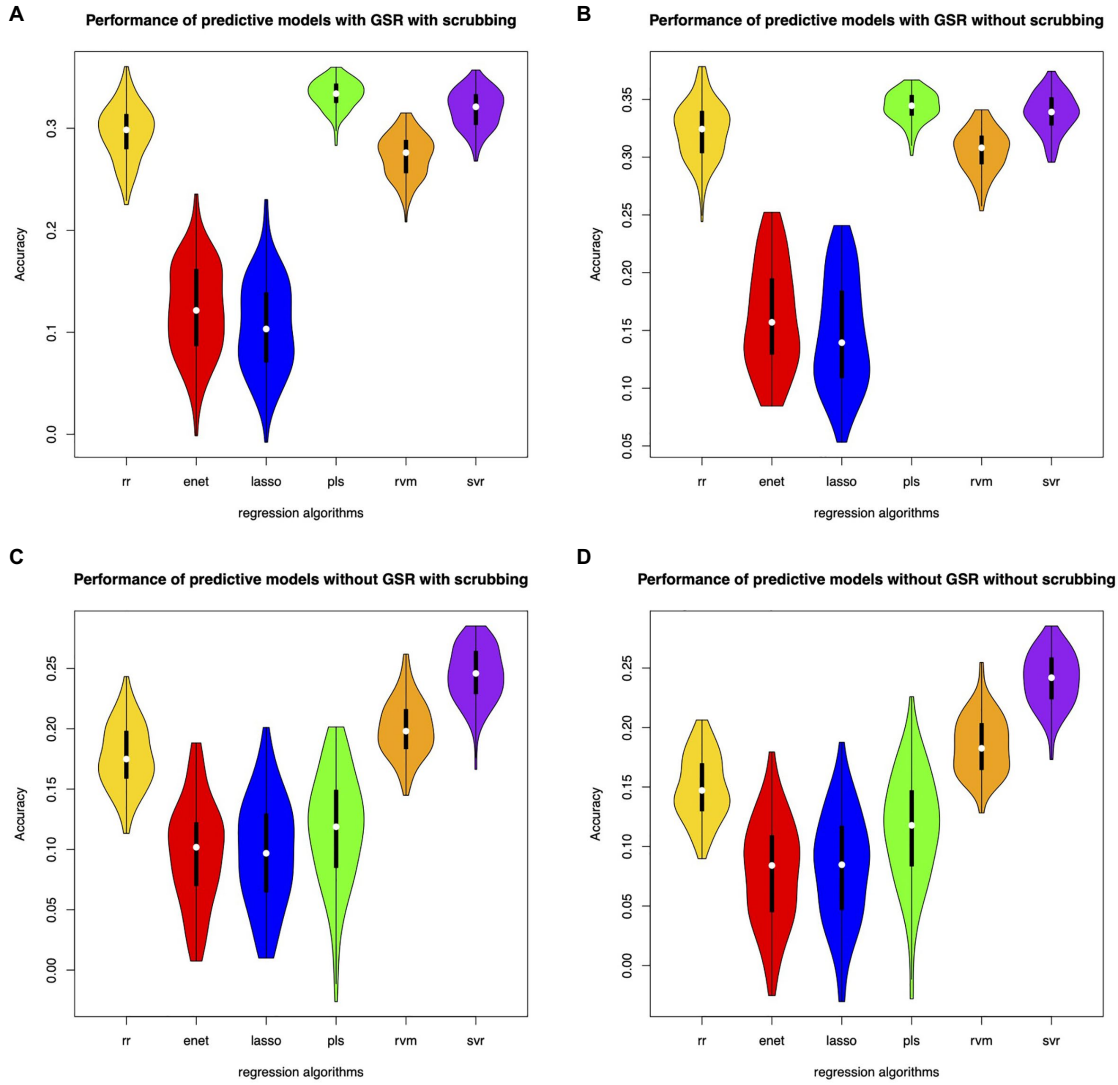
Performance of the predictive models with classical feature selection (*p*<0.05). **(A)** denotes performance of the predictive models with GSR and scrubbing. **(B)** denotes performance of the predictive models with GSR and without scrubbing. **(C)** denotes performance of the predictive models without GSR and with scrubbing. **(D)** denotes performance of the predictive models without GSR and scrubbing.

**Figure 3:**
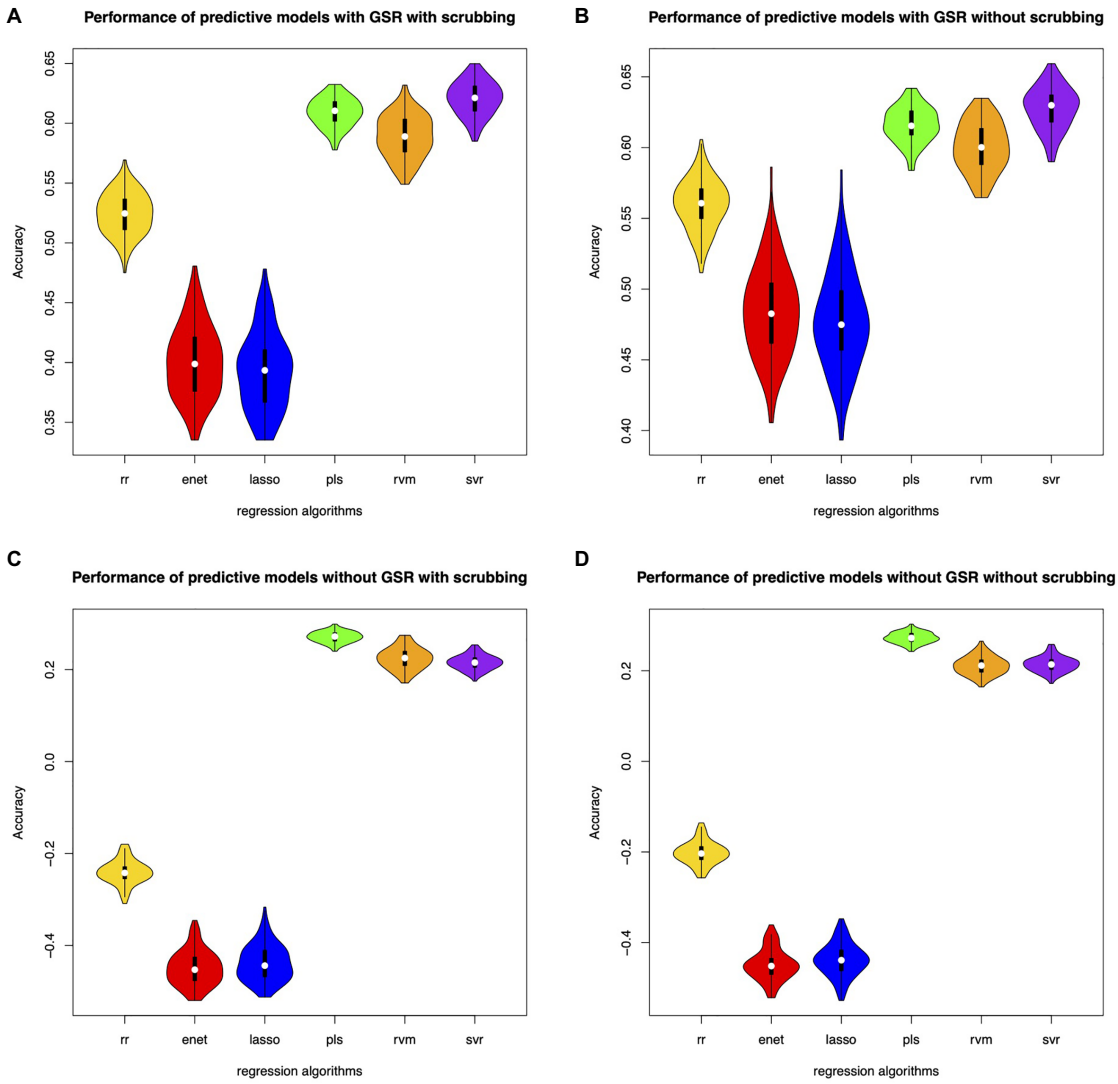
Performance of the predictive models with classical feature selection (*p*<0.05 and *r*>0). **(A)** denotes performance of the predictive models with GSR and scrubbing. **(B)** denotes performance of the predictive models with GSR and without scrubbing. **(C)** denotes performance of the predictive models without GSR and with scrubbing. **(D)** denotes performance of the predictive models without GSR and scrubbing.

**Figure 4:**
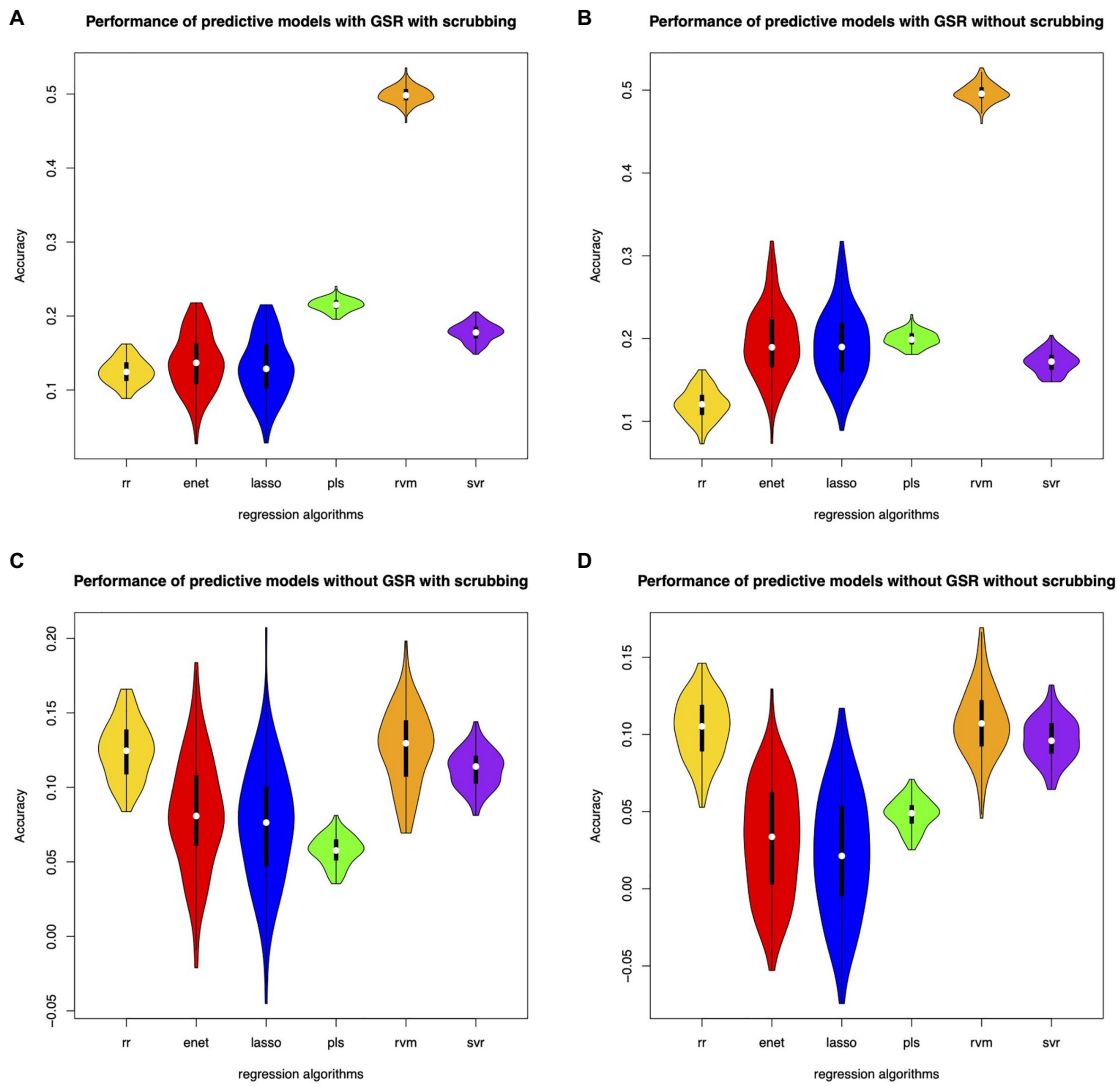
Performance of the predictive models with covariance-based feature selection. **(A)** denotes performance of the predictive models with GSR and scrubbing. **(B)** denotes performance of the predictive models with GSR and without scrubbing. **(C)** denotes performance of the predictive models without GSR and with scrubbing. **(D)** denotes performance of the predictive models without GSR and scrubbing.

**Figure 5:**
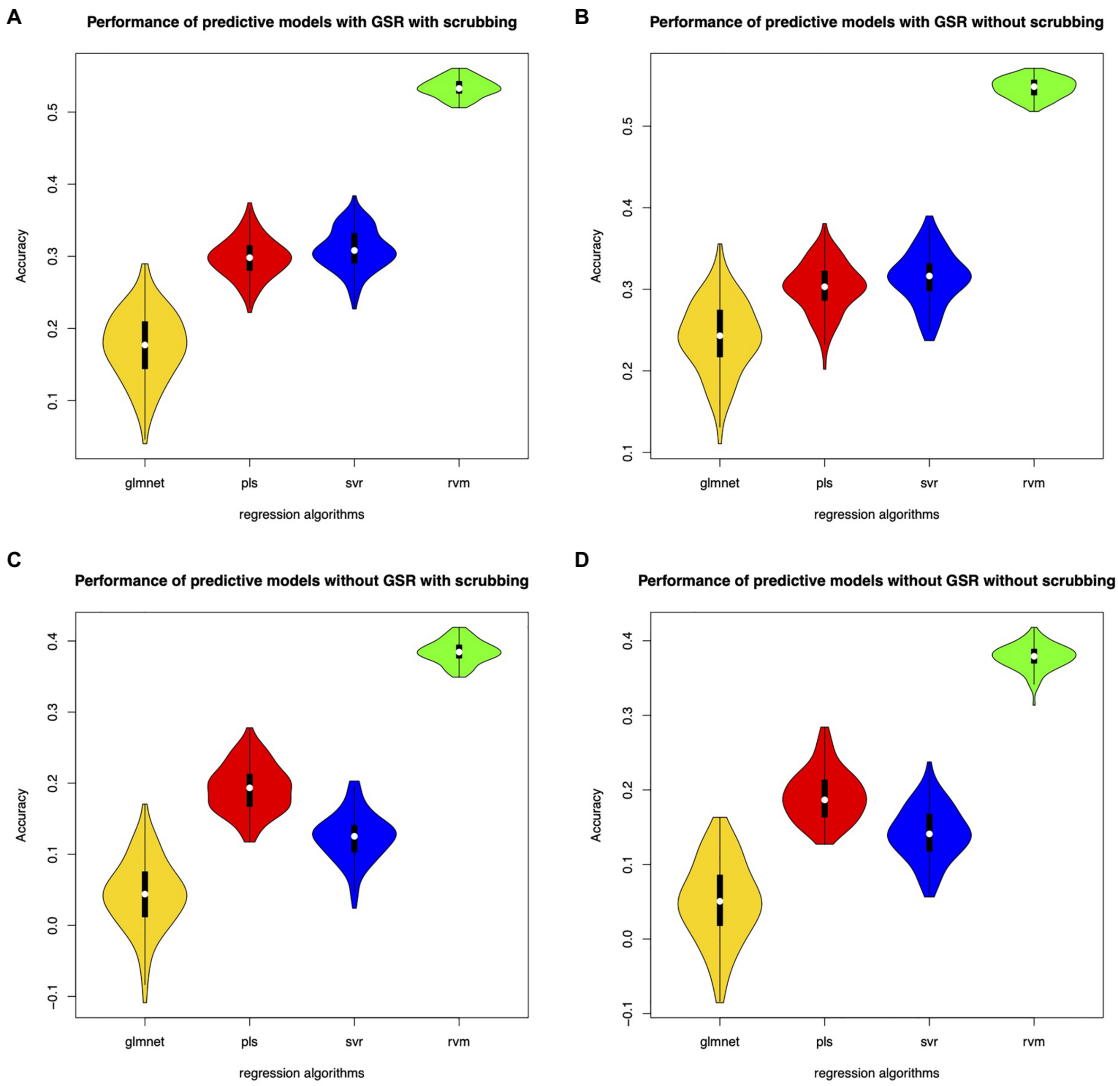
Performance of the predictive models with fine-tuned parameters. **(A)** denotes performance of the predictive models with GSR and scrubbing. **(B)** denotes performance of the predictive models with GSR and without scrubbing. **(C)** denotes performance of the predictive models without GSR and with scrubbing. **(D)** denotes performance of the predictive models without GSR and scrubbing.

**Figure 6:**
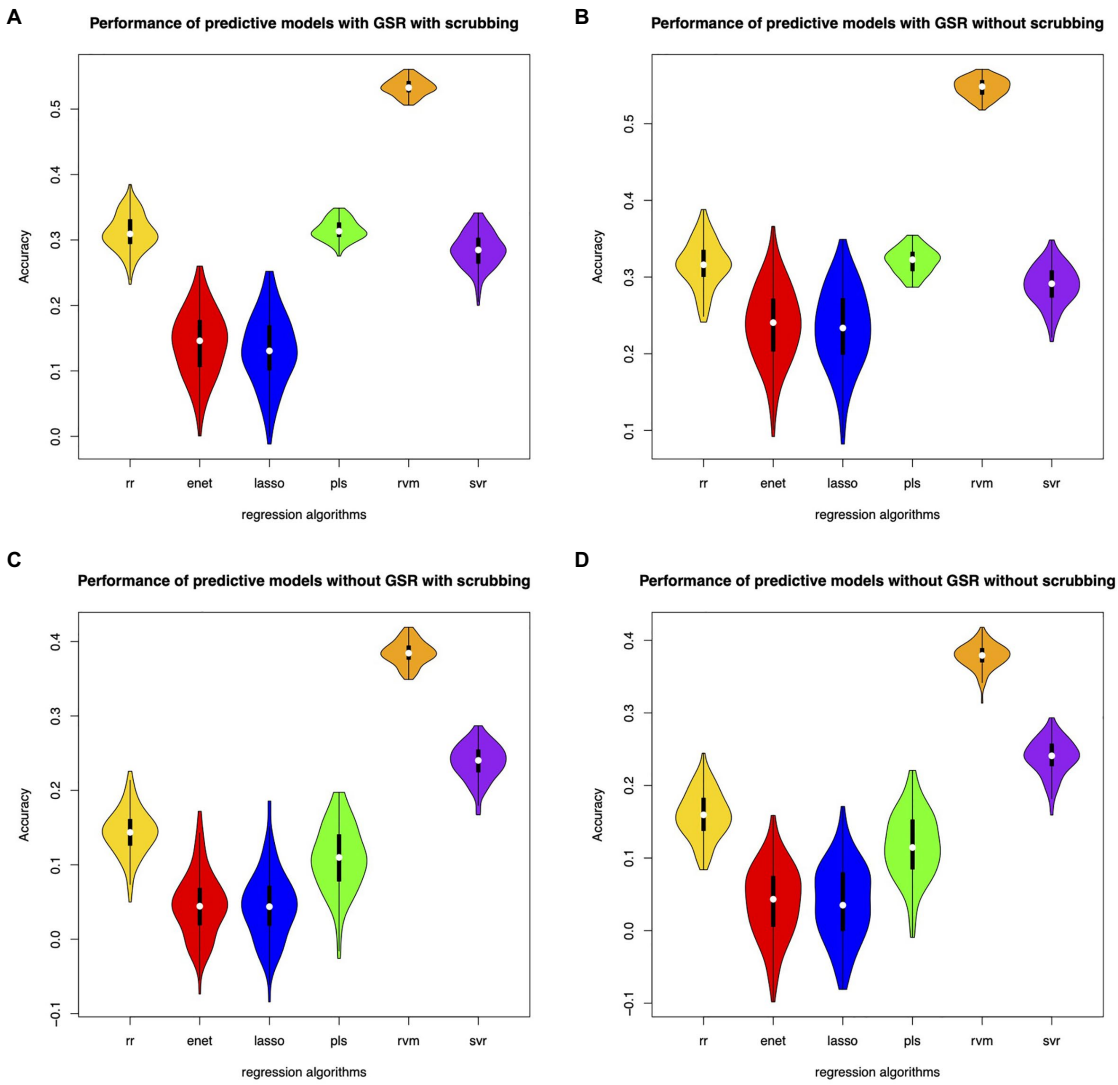
Performance of the predictive models with the mRMR feature selection. **(A)** denotes performance of the predictive models with GSR and scrubbing. **(B)** denotes performance of the predictive models with GSR and without scrubbing. **(C)** denotes performance of the predictive models without GSR and with scrubbing. **(D)** denotes performance of the predictive models without GSR and scrubbing.

Different feature selection methods and regression algorithms have impacts on the performance of the predictive models. Figure 2 shows the performance of the predictive models based on classical feature selection (*p*<0.05). Figure 3 shows the performance of the predictive models based on classical feature selection (*p*<0.05, *r*>0). Figure 4 shows the performance of the predictive models based on covariance feature selection. Figure 5 shows the performance of the predictive models based on fine-tuning of the regression algorithms. Figure 6 shows the performance of the predictive models based on mRMR feature selection. Table 1 shows the performances of predictive models based on classical feature selection with GSR and scrubbing. Table 2 shows the performances of predictive models based on mRMR with GSR and scrubbing. The CPM-based models with GSR and scrubbing can achieve a mean accuracy of 0.31. The best predictive models can achieve a total accuracy of 0.56 based on mRMR and RVM. The PLS also exhibits predictive powers. The PLS based on mRMR can achieve a total accuracy of 0.34.

**Table 1:** Performance of predictive models based on classical feature selection with GSR and scrubbing.

Algorithms	*r*	MAE	RMSE
RR	0.3 ± 0.03	5.95 ± 0.06	6.9 ± 0.06
ENET	0.12 ± 0.05	6.5 ± 0.14	7.6 ± 0.15
LASSO	0.11 ± 0.05	6.57 ± 0.14	7.74 ± 0.16
PLS	0.33 ± 0.01	5.83 ± 0.05	6.91 ± 0.05
RVM	0.27 ± 0.02	6.01 ± 0.05	6.97 ± 0.05
SVR	0.32 ± 0.02	5.94 ± 0.04	6.85 ± 0.04

**Table 2:** Performance of predictive models based on mRMR with GSR and scrubbing.

Algorithms	*r*	MAE	RMSE
RR	0.31 ± 0.03	5.92 ± 0.07	6.87 ± 0.07
ENET	0.14 ± 0.05	6.52 ± 0.19	7.77 ± 0.2
LASSO	0.13 ± 0.05	6.6 ± 0.21	7.88 ± 0.22
PLS	0.32 ± 0.02	5.9 ± 0.06	6.98 ± 0.06
RVM	0.53 ± 0.01	5.42 ± 0.03	6.28 ± 0.04
SVR	0.28 ± 0.03	6.12 ± 0.03	6.98 ± 0.03

The predictive models with GSR outperform that without GSR. Figures 2A,B, Figures 3A,B, Figures 4A,B, Figures 5A,B as well as Figures 6A,B show the performance of the predictive models with GSR. Figures 2C,D, Figures 3C,D, Figures 4C,D, Figures 5C,D, as well as Figures 6C,D show the performance of the predictive models without GSR. The performance of the predictive models with GSR is significantly higher than that without GSR.

The predictive models without scrubbing outperform those with scrubbing. Figures 2A,C, Figures 3A,C, Figures 4A,C, Figures 5A,C as well as Figures 6A,C show the performance of the predictive models with scrubbing. Figures 2B,D, Figures 3B,D, Figures 4B,D, Figures 5B,D, as well as Figures 6B,D show the performance of the predictive models without scrubbing. The performance of predictive models with scrubbing is a little lower than that without scrubbing.

In addition, the predictive models without fine-tuning (Figure 6) outperform that with fine-tuning (Figure 5). The positive weighted features significantly improve the performance of the regression models with GSR, but remarkably reduce the performance of the regression models without GSR, as indicated in Figure 3.

### Predictive Patterns Related to Inattention

**Figure 7:**
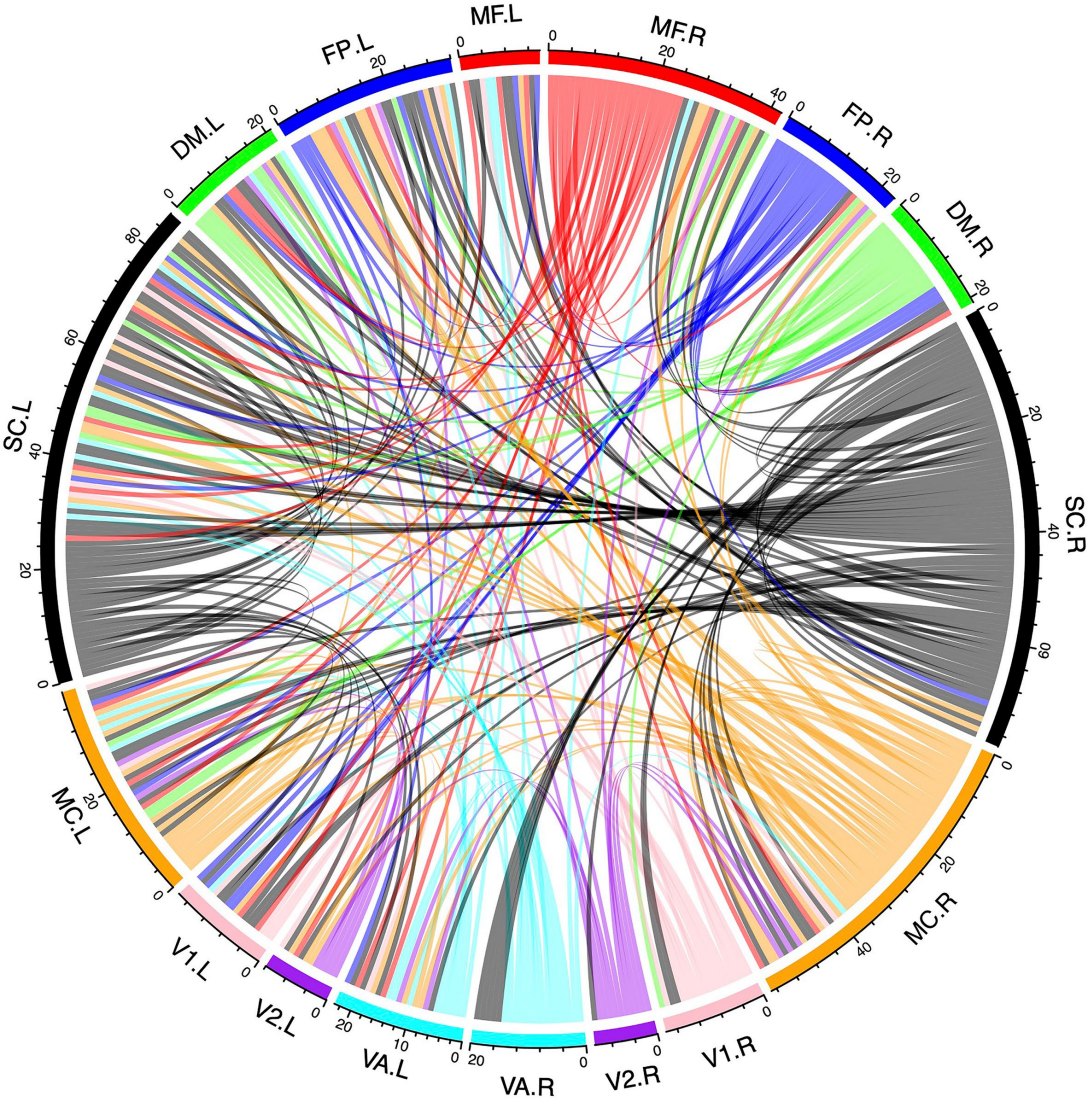
Predictive patterns of phase synchrony for inattention. MF stands for the medial frontal network. FP represents the frontoparietal network. DM means the default mode network. SC denotes the subcortical-cerebellum network. MC represents the motor cortex network. V1 denotes the visual I network. V2 denotes the visual II network. VA stands for the visual association network.

Figure 7 shows the predictive patterns related to inattention based on the mRMR feature selection with GSR and scrubbing. The 268 nodes are divided into 8 functional systems according to a previous study (Finn et al., 2015). The 8 functional systems are named as the medial frontal (MF) network, frontoparietal (FP) network, default mode (DM) network, subcortical-cerebellum (SC) network, motor cortex (MC) network, visual I (V1) network, visual II (V2) network, and visual association (VA) network. With 100 runs of 10-fold feature selection procedures, 1,000 arrays of most predictive features are selected as important attributes. Only features that appeared more than 900 times are displayed in Figure 7. The most predictive brain regions are located in the bilateral SC network. The second predictive brain regions are located in the bilateral MC network. The right MF network is more predictive than the left MF network. The DM network and visual networks are less predictive than other networks. Both intra- and inter-hemisphere connections are found for inattention estimation.

## Discussion

In this paper, we applied several feature selection methods and six regression algorithms to build predictive models for inattention estimation using phase synchrony. The effects of different preprocessing steps (i.e., GSR, scrubbing) were considered in computing phase synchrony. We found that the RVMs based on mRMR features significantly improve the performance of inattention estimation from resting state phase synchrony. In addition, we also found that GSR significantly enhanced the relationships between phase synchrony and inattention. Furthermore, the predictive patterns were discovered using mRMR methods. In summary, we proposed a novel framework for inattention estimation from phase synchrony, which could be supplementary biomarkers for predictive models.

The performance of regression models was related to several procedures in inattention estimation. First, the feature selection methods might affect the accuracy of prediction. The features selected by conventional correlation coefficients were univariate attributes, which did not consider the relationships among the raw features. The significant inattention-correlated features with positive weights (*p*<0.05 and *r*>0) can improve the performance of regression models but were dependent on GSR procedures. The performance of covariance-based feature selection was lower than that of conventional correlation-based models, since the covariance-based features might not be the significantly inattention-correlated. To overcome this limitation, mRMR was proposed to select multivariate features (Ding and Peng, 2005). The selected features significantly improved the performance of inattention estimation. Second, the regression algorithms also affect the performance of predictive models. We found that in addition to RVM, the PLS was an alternative algorithm for inattention estimation, which was consistent with previous findings (Yoo et al., 2017). Specially, we found RVMs based on mRMR features outperformed the other methods. The results indicated that the fine-tuning procedure does not improve the performance of the regression models. The poor performance of the fine-tuning might be caused by the 10-fold cross-validation procedures, since the training samples were different among the cross-validations. Of note, the RVM exhibited the best performance using automatic fine-tuning, implying that the sigma parameter for RVM was robust for different datasets. Third, the different preprocessing steps significantly affect the prediction. GSR significantly enhanced the relationships between phase synchrony and inattention. Scrubbing had little effect on the final results. The results suggested that GSR should be considered in brain-behavioral prediction task (Li et al., 2019a). Fourth, the cross-validations might have effect on the performance of prediction tasks. Here, 100 runs of 10-fold cross-validations were performed to evaluate the predictive models. The correlation coefficients were reliable and the MAE values were also stable, suggesting the robustness of the predictive models. In this paper, we applied different algorithms to build predictive models for inattention. After comparing with different methods, we found that the mRMR-RVM strategy might be beneficial for inattention estimation from neuroimaging features.

Predictive patterns related to inattention were discovered using mRMR feature selection. The visual networks, default mode networks, medial frontal network, frontoparietal network, subcortical-cerebellum network, as well as motor cortex exhibited altered phase synchrony in patients with ADHD. The predictive connections in visual network and motor cortex suggested that the sensorimotor functions might be distinctive in ADHD (Zang et al., 2007). The altered connectivity patterns in medial frontal network and frontoparietal network might reflect the inattention mechanisms in ADHD (Tao et al., 2017). Previous studies found altered functional connectivity in default mode networks in ADHD, suggesting the abnormal resting state baseline activity in patients (Hoekzema et al., 2014). Decreased subcortical volumes were also found in ADHD compared to healthy controls (Lu et al., 2019). In this study, we found that the bilateral subcortical-cerebellum networks exhibited the most predictive phase synchrony patterns. We also found that the motor cortex had the second predictive brain regions. Both inter- and intra-hemisphere synchrony patterns were found to be related to inattention. In addition, the altered phase synchrony exhibited asymmetry patterns. Those findings implied that the whole brain phase synchrony was predictive to inattention estimation. In summary, this study provided a new way to decode the inattention using phase synchrony and mRMR feature selection, which might be beneficial for individual prediction of inattention.

This study has several limitations which should be solved in future studies. First, the dynamic properties of functional connectivity remain unexplored for inattention. Novel feature extraction methods for dynamic phase synchrony should be investigated for inattention estimation. Second, the performance of the inattention estimations should be improved with novel feature selection methods and regression algorithms. Third, the mRMR features could not reflect the positive or negative correlations between phase synchrony and inattention. Fourth, the regression models should be tested using an independent dataset, although the regression models were well-validated using 100 runs of 10-fold cross-validations. Fifth, there were different MRI protocols for the samples, which should be scanned with the same MRI scanner and parameters. In summary, the feature extraction models, feature selection methods, regression algorithms, and testing procedures should be improved to enhance the performance and the generalization ability of the regression models for individual inattention estimation.

## Conclusion

This paper applied different algorithms to build the predictive models for inattention from resting state fMRI. We also analyzed the impacts of different preprocessing steps on the predictive models. The RVMs based on mRMR features significantly improve the performance of inattention estimation from resting state phase synchrony. We also found that PLS might be an alternative method for brain-behavioral prediction tasks. In addition, the GSR strengthens the relationships between neuroimaging features and behavioral scores. In summary, we proposed a unified framework for brain-behavioral models based on phase synchrony. We also found an optimized strategy named mRMR-RVM for inattention estimation.

## Data Availability Statement

Publicly available datasets were analyzed in this study. These data can be available at: http://fcon_1000.projects.nitrc.org/indi/adhd200.

## Ethics Statement

The studies involving human participants were reviewed and approved by the Ethics Committee of Peking University. Written informed consent to participate in this study was provided by the participants’ legal guardian/next of kin.

## Author Contributions

X-HW and LL contributed to conception and design of the study and wrote the first draft of the manuscript. X-HW performed the statistical analysis. All authors contributed to manuscript revision, read, and approved the submitted version.

## Funding

This research was supported in part by the National Key R&D Program of China under grant no. 2018YFA0701702 and the National Natural Science Foundation of China (62071158).

## Conflict of Interest

The authors declare that the research was conducted in the absence of any commercial or financial relationships that could be construed as a potential conflict of interest.

The handling editor declared a past co-authorship with one of the authors LL.

## Publisher’s Note

All claims expressed in this article are solely those of the authors and do not necessarily represent those of their affiliated organizations, or those of the publisher, the editors and the reviewers. Any product that may be evaluated in this article, or claim that may be made by its manufacturer, is not guaranteed or endorsed by the publisher.

## Footnotes

^1^ http://fcon_1000.projects.nitrc.org/indi/adhd200

^2^ https://cran.r-project.org/web/packages/praznik

^3^ https://cran.r-project.org/web/packages/e1071/index.html

^4^ https://CRAN.R-project.org/package=textir

^5^ https://www.rdocumentation.org/packages/kernlab/versions/0.9-29

^6^ https://cran.r-project.org/web/packages/glmnet/index.html

^7^ https://github.com/YaleMRRC/CPM

^8^ https://topepo.github.io/caret/index.html

^9^ https://cran.r-project.org/package=RVAideMemoire
